# *Bacillus cereus* Spores Release Alanine that Synergizes with Inosine to Promote Germination

**DOI:** 10.1371/journal.pone.0006398

**Published:** 2009-07-28

**Authors:** Tetyana Dodatko, Monique Akoachere, Stefan M. Muehlbauer, Forrest Helfrich, Amber Howerton, Christian Ross, Vicki Wysocki, Jürgen Brojatsch, Ernesto Abel-Santos

**Affiliations:** 1 Department of Biochemistry, Albert Einstein College of Medicine, Bronx, New York, United States of America; 2 Department of Chemistry, University of Nevada, Las Vegas, Las Vegas, Nevada, United States of America; 3 Department of Microbiology and Immunology, Albert Einstein College of Medicine, Bronx, New York, United States of America; 4 Department of Chemistry and Biochemistry, University of Arizona, Tucson, Arizona, United States of America; 5 School of Life Science, University of Nevada, Las Vegas, Las Vegas, Nevada, United States of America; Charité-Universitätsmedizin Berlin, Germany

## Abstract

**Background:**

The first step of the bacterial lifecycle is the germination of bacterial spores into their vegetative form, which requires the presence of specific nutrients. In contrast to closely related *Bacillus anthracis* spores, *Bacillus cereus* spores germinate in the presence of a single germinant, inosine, yet with a significant lag period.

**Methods and Findings:**

We found that the initial lag period of inosine-treated germination of *B. cereus* spores disappeared in the presence of supernatants derived from already germinated spores. The lag period also dissipated when inosine was supplemented with the co-germinator alanine. In fact, HPLC-based analysis revealed the presence of amino acids in the supernatant of germinated *B. cereus* spores. The released amino acids included alanine in concentrations sufficient to promote rapid germination of inosine-treated spores. The alanine racemase inhibitor D-cycloserine enhanced germination of *B. cereus* spores, presumably by increasing the L-alanine concentration in the supernatant. Moreover, we found that *B. cereus* spores lacking the germination receptors *gerI* and *gerQ* did not germinate and release amino acids in the presence of inosine. These mutant spores, however, germinated efficiently when inosine was supplemented with alanine. Finally, removal of released amino acids in a washout experiment abrogated inosine-mediated germination of *B. cereus* spores.

**Conclusions:**

We found that the single germinant inosine is able to trigger a two-tier mechanism for inosine-mediated germination of *B. cereus* spores: Inosine mediates the release of alanine, an essential step to complete the germination process. Therefore, *B. cereus* spores appear to have developed a unique quorum-sensing feedback mechanism to monitor spore density and to coordinate germination.

## Introduction

*B. cereus* and *B. anthracis* form dormant spores that survive harsh environmental conditions. Upon encountering a suitable environment, these spores germinate into their vegetative form [1], [2]. Binding of specific germinants including amino acids, nucleosides, and other small molecules to their cognate membrane receptors (Ger proteins) is believed to initiate the germination process [3], [4]. Ger receptors are essential for germination and encoded as tricistronic operons [5]. Following the activation of these receptors, *B. cereus* spores release dipicolinic acid (DPA), calcium, and amino acids [4], [6]. Subsequently, the cores of the spores becomes hydrated, and the spore cortex and spore-specific proteins are hydrolyzed [7], [8], [9]. Amino acids are released into the extracellular milieu following germination from an internal pool and from protein degradation [6], [10], [11]. Approximately 30 min after addition of germinants, the newly germinated cells start to divide [4], [12].

While *B. cereus* and *B. anthracis* spores recognize nucleosides and amino acids as germinants, the species respond differently to these germinants [13], [14], [15]. While *B. cereus* 569 spores are able to germinate in the presence of a single germinant (inosine), *B. anthracis* spores require either a combination of inosine and an amino acid, or two different amino acids in order to germinate [15], [16]. GerI and GerQ receptors have been linked to inosine-mediated germination of *B. cereus* 569 spores [13], [17]. *B. cereus* spores lacking the GerQ receptor are unable to germinate in the presence of inosine alone, while those lacking the GerI receptor show reduced germination rates in the presence of inosine [13]. However, *gerI* and *gerQ*-deficient strains germinate efficiently in the presence of a combination of inosine and alanine [13], [14]. Thus, the presence of the second germinant alanine appears to compensate for the deficiency of *gerI* and *gerQ*-negative spores. While two Ger receptors have been linked to inosine-mediated germination in *B. cereus* [3], [13], [14], only one Ger receptor (GerH) has been linked to inosine in *B. anthracis* [15], [18]. GerI and GerH share high sequence homology (96%, 92%, and 89% identity for the A-, B-, and C-subunit, respectively), while the germination receptor GerQ is only minimally related to GerI and GerH receptors [3], [15].

We have previously demonstrated that *B. cereus* spores germinate with a time lag and non-linear kinetics when inosine is used as the sole germinant [17]. This lag phase is greatly reduced when inosine is supplemented with alanine. Others and we have shown that numerous nucleoside analogs are able to germinate *B. cereus* spores when supplemented with alanine [3], [17], [19], [20]. Inosine, on the other hand, is the only nucleoside that efficiently germinates *B. cereus* spores when used in the absence of a co-germinant [17]. In contrast to *B. cereus* spores, germination of *B. anthracis* spores requires the presence of two germinants such as inosine and alanine [16]. These germinants bind to *B. anthracis* spores with strong cooperativity [16].

In this study, we analyzed inosine-mediated germination of *B. cereus* 569 spores. We demonstrated that *B. cereus* release amino acids and specifically alanine when germination is triggered by inosine as the sole germinant. Amino acid release following inosine exposure required the presence of both GerI and GerQ receptors. We provide evidence that alanine release is essential for germination of *B. cereus* spores treated with a single germinant. We also found that alanine release enhances the germination kinetics of inosine-treated spores, and speculate that alanine release serves as a positive feedback loop to bring about spore germination.

## Results

### Conditioned supernatant from germinated *B. cereus* spores increases inosine-mediated germination rate of these spores

**Figure 1:**
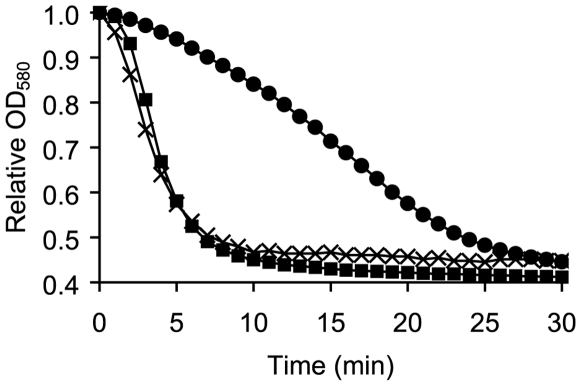
Enhanced germination rate of B. cereus spores germinated in conditioned supernatants. Wild-type *B. cereus* spores were germinated with a 0.2 mM inosine solution (•) or in conditioned supernatants containing 0.2 mM inosine (▪). *B. cereus* spores were also germinated with 0.2 mM inosine and 20 µM alanine (X).

We recently described that *B. cereus* 569 spores germinate with a time lag when inosine is used as the sole germinant [17]. This lag phase is significantly reduced and germination rates increase considerably when inosine is supplemented with alanine (1). Following the lag phase, *B. cereus* spores treated with inosine germinate with non-linear kinetics. We hypothesized that cofactors released from germinating spores during the lag phase enhance germination kinetics. To test this, we treated *B. cereus 569* spores with 0.2 mM inosine, and collected supernatants 30 min post-inosine exposure. The conditioned supernatants derived from germinated spores were then added to fresh *B. cereus* spores. As shown in Fig. 1, conditioned supernatants collected from germinated spores significantly accelerated germination of fresh *B. cereus* spores. The lag phase was greatly shortened in the presence of conditioned supernatants, and the resulting germination kinetics resembled those obtained when 0.2 mM inosine was supplemented with 20 µM alanine (Fig. 1). Heat-treated (90°C for 15 min) or micro-filtrated (5 kDa MWCO) conditioned supernatants showed similar acceleration of the germination rate as untreated conditioned supernatants (data not shown). Together, these findings indicate that *B. cereus* spores release low molecular weight and heat-stable germination cofactors that promote inosine-mediated germination.

### The potency of the conditioned supernatants is dependent on inosine and spore concentrations

**Figure 2:**
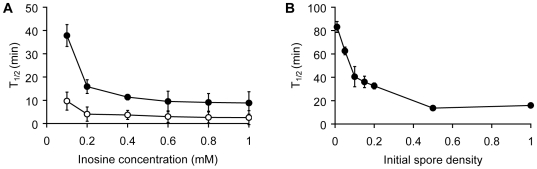
Germination rates as a function of inosine and spore concentrations. (A) Wild-type *B. cereus* spores were germinated with increasing inosine concentrations (•). *B. cereus* spores were also germinated in conditioned supernatants collected 30 min after exposure to increasing inosine concentrations (○). T_1/2_ values were calculated for each one of the spore sample germinated in inosine and conditioned buffer and plotted against the initial inosine concentration. T_1/2_ values correspond to the time it takes to reach half-maximal values. (B) *B. cereus* spores were resuspended at increasing optical densities (OD_580_) and germinated in the presence of 0.2 mM inosine. After 30 min, conditioned supernatants were isolated from the germinated spores. Fresh spore aliquots were resuspended in the conditioned supernatants to an optical density of 1. Germination curves were monitored as described above. T_1/2_ values were calculated from each spore sample germinated in conditioned buffer and plotted against the initial spore optical densities.

To determine conditions that promote germination, *B. cereus* spores were germinated at different spore densities, or in the presence of increasing inosine concentrations. Conditioned supernatants collected 30 min post-germination were added to fresh spores and T_1/2_ values were determined. T_1/2_ values represent the time point when the optical density has reached 50% of its final value. As expected, germination T_1/2_ times decreased with increasing inosine concentrations (Fig. 2A). Similarly, the potency of conditioned supernatants increased when they were harvested from spores germinated at increasing spore concentrations as indicated by decreased T_1/2_ values (Fig. 2B).

**Figure 3:**
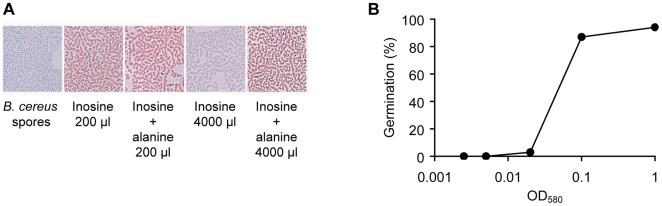
*B. cereus* spore germination at low spore concentrations. (A) *B. cereus* spores were diluted in 10 or 4000 ml (OD_580_ of 1 or 0.0025, respectively) of germination buffer supplemented with 0.2 mM inosine and 0 or 40 µM alanine. Thirty min post-inosine exposure, spores were collected by centrifugation, and pellets were treated with malachite green to stain resting spores and safranin-O to stain germinated cells. Samples were placed under a microscope and a field selected at random. (B) *B. cereus* spores were diluted in germination buffer (OD_580_ of 1 to 0.0025) in the presence of 0.2 mM inosine. Stained samples were placed under a light microscope and the amount of resting spores and germinated cells were counted on three different fields selected at random. The percentage of germinated spores was plotted against the initial spore optical density.

We also tested whether germination of *B. cereus* spores by inosine alone required a specific spore density. Towards this, we diluted 10 ml of spores in increasing volumes germination buffer containing 0.2 mM inosine. Following continuous shaking at 37°C, germination was determined by microscopy using a modified Wirtz-Conklin stain [21]. This protocol stains resting and germinated spores green and red, respectively. Strikingly, germination of *B. cereus* spores was impaired at high dilutions, as less than 3% of spores germinated when diluted to ODs ranging from 0.0025 to 0.02 (Fig. 3A and 3B). On the other hand, *B. cereus* spores germinated efficiently (>87%) at high concentration (OD of 0.1 and 1). As expected, *B. cereus* spores germinated efficiently when inosine was supplemented with 40 µM alanine regardless of spore density (Fig. 3A). These results indicate that inosine-mediated germination of *B. cereus* spores requires a minimal spore density.

### Dipicolinic acid (DPA) release cannot account for germination acceleration

**Figure 4:**
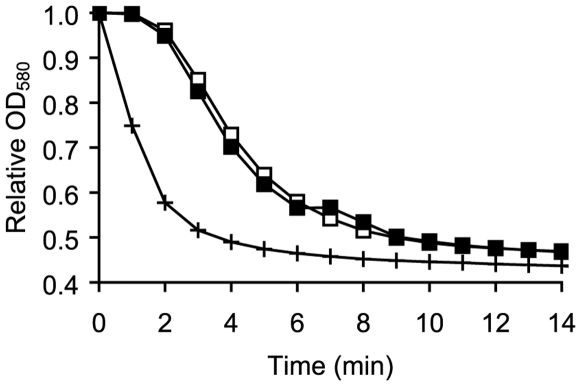
*B. cereus* spore germination in the presence of Ca-DPA. (A) Wild-type *B. cereus* spores were germinated in the presence of 0.2 mM inosine (□). *B. cereus* spores were also germinated in the presence of conditioned supernatants containing 0.2 mM inosine (+). Spores were also germinated with 0.2 mM inosine supplemented with 0.18 mM Ca-DPA (▪).

A release of DPA has been linked to increased germination efficiencies, presumably through the activation of cortex-lytic enzymes [22]–[25]. While *B. cereus* spores germinate in the presence of 60 mM extracellular calcium-DPA [23], [24], [25], the final DPA concentration in the conditioned medium of germinated *B. cereus* spores was only 0.18 mM [26]. To test whether released DPA and/or calcium could account for the enhanced germination kinetics observed in the presence of conditioned supernatants, we exposed spores to 0.2 mM inosine supplemented with Ca-DPA (Fig. 4). As a control, we germinated spores in the presence of inosine alone. The presence of 0.18 mM Ca-DPA did not accelerate inosine-mediated germination (Fig. 4), suggesting that DPA is not a co-germinant in this process.

### D-cycloserine improves the efficiency of conditioned media to germinate *B. cereus* spores

**Figure 5:**
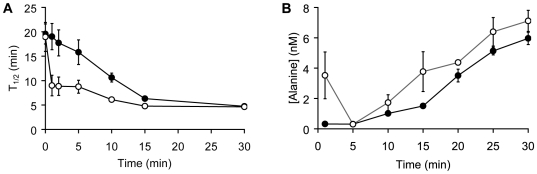
Kinetics of auto-inducer release. (A) Wild-type *B. cereus* spores were germinated with 0.2 mM inosine supplemented with 0 or 1 mM D-cycloserine. Conditioned supernatants were collected at different time points post-inosine addition. Fresh *B. cereus* spores were resuspended in the conditioned supernatants that contained inosine and either 0 (•) or 1 (○) mM D-cycloserine and germination curves monitored as described above. T_1/2_ values were plotted against the time point of supernatant collection. (B) Wild-type *B. cereus* spores were germinated with 0.2 mM inosine supplemented with either 0 (•) or 1 mM D-cycloserine (○). The conditioned supernatants were collected at different intervals post-inosine addition and amino acids were derivatized with isobutyl groups. Total alanine concentration was determined by comparison with known alanine standards.

We also determined the effect of increasing incubation times on the potency of the harvested supernatants on inosine-mediated germination of *B. cereus* spores. As expected, germination efficiency of harvested supernatants increased with incubation time: germination was most efficient using supernatant collected 30 min post-inosine exposure (Fig. 5A). No increase in germination rate was observed when conditioned media was collected within 5 min of inosine exposure. Taken together, the potency of conditioned media increased with inosine and spore concentrations, as well as with longer incubation times.

We subsequently tested whether altering levels of the co-germinant alanine changes germination kinetics of inosine-treated *B. cereus* spores. Bacterial spores contain two alanine isomers: L-alanine and D-alanine. L-alanine has been shown to promote germination of multiple bacterial spores [16], [17], [27], [28], while D-alanine has been described to block germination [29]. *B. cereus* spores express the endogenous enzyme alanine racemase on the surface. Alanine racemase is able to convert the activating L-alanine into the inhibitory D-alanine [30]. Inhibition of alanine racemase has been shown to increase L-alanine-mediated germination rates. The presence of D-cycloserine significantly increased the germination rates of inosine-treated *B. cereus* spores compared to spores exposed to inosine only (Fig. 5A). The increased germination kinetics further implicates L-alanine in germination of inosine-treated *B. cereus* spores.

### Germinated *B. cereus* spores release amino acids

Since addition of alanine mimics the effect of conditioned media on inosine-treated spores (Fig. 1), we determined the concentration of released amino acids in the conditioned *B. cereus* supernatants using 7-amino, 4-methylcoumarin (7-AMC) labeling. 7-AMC is a fluorescent dye that has been used to label amino acids and peptides [31], [32]. The concentration of amino acids in *B. cereus* conditioned supernatants was approximately 80 µM as determined by 7-AMC-labeling. As expected, no amino acids were detected in the supernatant of *B. anthracis* spores treated with inosine only.

Following HPLC separation and mass spectrometry of 7-AMC-labeled supernatants, we detected alanine, glycine, leucine, threonine, and serine as major compounds in the supernatant of germinated *B. cereus* spores (Table 1). The final concentration of each amino acid ranged from 5 to 20 µM. An amino acid standard mixture containing alanine, glycine, leucine, threonine, and serine showed the same elution profile as compounds identified in the conditioned supernatant from *B. cereus* spores.

**Table 1:** 7-AMC adducts detected in *wt B. cereus* conditioned supernatants.

Amino acid	Concentration (μM)	Expected MW (Da)	Observed MW (Da)
Alanine	20	246.266	246.135
Serine	18	262.268	262.193
Leucine	13	288.347	288.156
Glycine	11	232.240	232.111
Threonine	5	276.293	276.242

Wild type *B. cereus* 569 spores were resuspended in 200 µl TMB buffer to OD_580_ = 1. Spores were treated with 0.2 mM inosine and supernatants were collected 30 min post-inosine addition. Collected supernatants were labeled with 7-AMC. 7-AMC adducts sere separated by RP-HPLC and identified by mass spectrometry. Concentrations were calculated by fluorescence spectroscopy.

To determine whether the amino acid released could act as a co-germinant with inosine to accelerate spore germination, we treated spores with inosine and each one of the amino acid identified above. Consistent with our findings, only alanine (data not shown) was able to synergize with inosine to increase the germination rate. Germination acceleration was identical at L-alanine concentrations between 8 µM and 20 µM.

To determine the kinetics of alanine release, we collected supernatants from spores germinated at different time points after inosine addition. These supernatants were derivatized with isobutyl groups to enhance fragmentation for quantitative analysis by tandem mass spectrometry. As expected, the alanine concentration increased continuously during the germination process (Fig. 5B). In fact, the alanine concentration increased almost 100-fold during the first 25 min of germination (Fig. 5B). Furthermore, the kinetics of alanine accumulation in the supernatant of germinated spores follows the same trend as the increase in germination rate as a function of incubation time (Fig. 5A). As expected, D-cycloserine did not increase the total amount of alanine in the supernatant, and similar amount of alanine was released in the presence and absence of D-cycloserine (Fig. 5B). These findings suggest that the enhanced germination in the presence of D-cycloserine is not due to increased levels of total alanine, but rather due to increased levels of L-alanine.

In contrast to *B. cereus* spores, the concentrations of inosine and alanine did not change in the supernatants from *B. anthracis* spores following germination with inosine and alanine (data not shown). These results further support the notion that amino acid release is restricted to germinating *B. cereus*, and does not occur in *B. anthracis* spores.

#### Concentration of free alanine in *B. cereus* spores

An aliquot of *B. cereus* spores was resuspended under conditions identical for the quantification of alanine in the supernatant (see above). Enough free alanine was obtained from the ungerminanted spores to yield a final concentration of 4.8 µM.

### *ΔgerI* and *ΔgerQ B. cereus* spores fail to release amino acids

**Figure 6:**
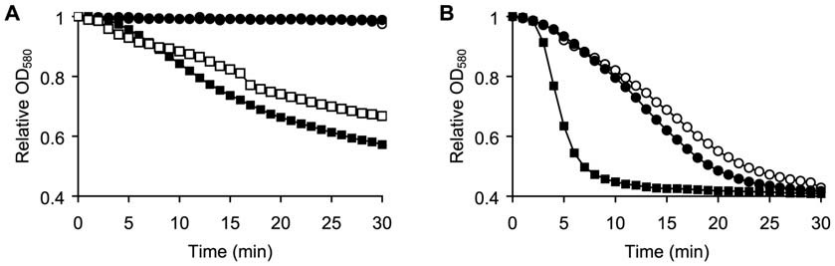
*B. cereus* spore germination in the presence of conditioned supernatants from *ΔgerQ* or wild type spores. (A) Wild type or Δ*gerQ B. cereus* spores were treated with 0.2 mM inosine. Conditioned supernatants were collected 30 min post-inosine addition. Fresh Δ*gerQ B. cereus* spores were germinated in the supernatants obtained from wild type (▪) or Δ*gerQ* (•) spores. Δ*gerQ B. cereus* spores were also treated with 0.2 mM inosine (○) and 0.2 mM inosine supplemented with 20 µM alanine (□). (B) Wild type and Δ*gerQ B. cereus* supernatants were individually prepared as described above. Fresh wild type *B. cereus* spores were resuspended in conditioned supernatants from wild type (▪) or Δ*gerQ* (•) *B. cereus* spores. Wild type *B. cereus* spores were also treated with 0.2 mM inosine (○).

The GerI and GerQ receptors of *B. cereus* are required for efficient germination in the presence of inosine [13], [14]. We found that *B. cereus* spores containing a deletion in the GerQ receptor gene (*ΔgerQ* spores) did not germinate in the presence of inosine as the sole germinant (Fig. 6A). However, *ΔgerQ* spores germinated efficiently when inosine was supplemented with alanine or with conditioned media from germinated wild-type *B. cereus* spores. In fact, the germination kinetics of *ΔgerQ* spores obtained with conditioned media were similar to those acquired with inosine and alanine (Fig. 6A). Our results are consistent with findings showing that *ΔgerQ* spores germinate normally when inosine is supplemented with alanine [13], [14]. These results indicate that the responsiveness to primary (inosine) and secondary (alanine) germinants is not compromised in *ΔgerQ* spores, and that these spores germinate normally in the presence of both germinants.

As predicted, conditioned supernatants harvested from inosine-treated *ΔgerQ B. cereus 569* spores had no significant effect on the germination rate of wild type or *ΔgerQ* spores (Fig. 6B). Similar results were observed with conditioned media isolated from inosine-treated *ΔgerI B. cereus 569* spores (data not shown). Subsequently, we tested whether *ΔgerQ* and *ΔgerI* spores are defective in their ability to secrete germination cofactors by using 7-AMC-labeling procedure as described above. As expected, inosine-treated *ΔgerQ* and *ΔgerI* spores did not release any amino acids (data not shown). It is reasonable to assume that the failure to release amino acids is responsible for the results obtained with *ΔgerQ* and *ΔgerI* spores treated with inosine only.

### Conditioned supernatants from *B. cereus* spores do not increase the germination rate of *B. anthracis* spores

In contrast to *B. cereus* spores, germination of *B. anthracis* spores requires at least two different germinants and does not occur in the presence of inosine only [16]. Intriguingly, conditioned media obtained from germinating *B. cereus* spores failed to germinate inosine-treated *B. anthracis* spores. Accordingly, 20 µM alanine, the alanine concentration found in the supernatant of germinated *B. cereus* spores, was sufficient to germinate *B. cereus* when used with inosine, but was insufficient to germinate *B. anthracis* spores (data not shown). *B. anthracis* spores, however, germinated efficiently when the alanine concentration was increased to 100 µM. Taken together, our findings suggest that inosine triggers the release of amino acids, most notably alanine, from *B. cereus* spores. This step appears to be required for completion of the germination process. This positive feedback loop appears to be mediated by GerI/GerQ receptors.

Taken together, we have demonstrated that *B. cereus* spores, in contrast to *B. anthracis* spores, are able to germinate in the presence of a single external germinant. We have shown that the single germinant inosine is able to trigger a feedback loop that results in the release of amino acids, presumably alanine. This amino acid release appears to be the second step required to complete the germination process.

## Discussion

Here we present multiple findings supporting the theory that alanine is released during *B. cereus* germination, and is required for germination of these spores in the presence of inosine as the sole germinant: 1) We found that inosine-treated *B. cereus* spores release alanine in sufficient concentrations to positively affect germination. The concentration of DPA, calcium, and other amino acids released from germinated spores, on the other hand, was too low to affect germination kinetics. 2) Blocking alanine racemase with D-cycloserine enhanced germination kinetics, consistent with L-alanine-mediated germination [33], [34], [35]. 3) Amino acid release was required for germination, as spores defective in amino acid release did not germinate in the presence of inosine as sole germinant. Taken together, our findings suggest that alanine is the major co-germinant released by *B. cereus* stimulated with inosine only.

We have demonstrated that the lag phase of germination observed in inosine-treated *B. cereus* spores is greatly reduced when inosine is supplemented with alanine or conditioned media. Our data suggests that this lag phase corresponds to the time it takes the inosine-activated spores to release amino acids/alanine in sufficient quantities to aid in spore germination. We have shown that *B. cereus* germination is significantly enhanced in the presence of D-cycloserine, which increases the concentration of active L-alanine. These findings mimic earlier studies demonstrating the enhancing effect of D-cycloserine on germination of *B. thuringiensis* spores in the presence of inosine [36].

Our findings are consistent with studies linking an increase in levels of endogenous amino acids with enhanced germination kinetics of inosine-treated *B. cereus* spores [37]. Moreover, increased spore density has been shown to enhance germination rates of different *Bacillus* species [38], supporting the notion that released germinants aid in the germination process.

*B. cereus* spores lacking GerI or GerQ receptors failed to germinate in the presence of inosine only. We found that *gerI* and *gerQ*-deficient spores did not release amino acids indicating that the defect was in the release of co-germinants. Moreover, *gerI* and *gerQ*-deficient spores germinated normally when inosine was supplemented with alanine or preconditioned supernatants derived from germinated *B. cereus* spores. Both receptors have been linked to inosine binding, however, the ability of *gerI* and *gerQ–*deficient spores to germinate efficiently in the presence of inosine and alanine indicates that recognition of these germinants is not impaired in these spores [14]. Intriguingly, *B. anthracis* does not release amino acids upon germination with inosine and alanine. Thus, inosine-mediated amino acid release seems to be a unique property of *B. cereus* 569 spores. We have shown that *gerI* and *gerQ*-negative *B. cereus* spores fail to release amino acids and to germinate in the presence of inosine. Since the B-subunit of germination receptors are related to bacterial amino acid exporter proteins [4], it is possible that GerI and GerQ receptors are directly involved in amino acid transport. It is also conceivable that these receptors stimulate amino acid transporters indirectly in inosine-treated spores. Our findings suggest that the mixture of exogenous inosine and released alanine activates secondary germination receptors that are presumably essential for the completion of the germination process.

Because *B. anthracis* spores do not release amino acids, they appear to require two germinants to bring about a successful germination [15], [18], [39], [40]. Having to simultaneously detect structurally different compounds might prevent *B. anthracis* spores, an obligate pathogen, from germinating outside a suitable host. Like *B. anthracis*, *B. cereus* efficiently germinates in the presence of two germinants. However, in addition to the *“two-germinant mode” B. cereus* has also developed a mechanism that allows it to germinate in the presence of a single germinant, provided that the spores have reached a certain density. It is conceivable that the alanine release provides *B. cereus* with a feedback loop to finish the germination process. This feedback loop requires a critical density of *B. cereus* spores for optimal germination, and might allow *B. cereus* to monitor spore density and to coordinate germination. Our findings suggest that *B. cereus* spores not only sense the environment for nutrients, but also for spore density.

Because *B. anthracis* spores do not release amino acids in the presence of inosine, germination of these spores is independent of their density. In fact, *B. anthracis* spores may actually use an opposite strategy. Conditioned media obtained from *B. anthracis* spores germinated with inosine and alanine inhibited germination of fresh *B. anthracis* spores. In this case, exogenous L-alanine was converted to D-alanine by the alanine racemase enzyme, thus resulting in an inhibitory conditioned supernatant [33], [34], [35]. The differences in strategies of *B. cereus* and *B. anthracis* spores might have evolved to take advantage of different environmental niches. *B. cereus*, unlike *B. anthracis*, is not an obligate pathogen. While *B. anthracis* germination outside the host would be detrimental for the pathogen, *B. cereus* might require less stringent conditions. Taken together, *B. cereus* spores appear to have developed a unique quorum-sensing mechanism to coordinate their germination processes.

## Materials and Methods

### Reagents and materials

Nucleosides were purchased from Sigma-Aldrich (St. Louis, MO). The *B. cereus 569* strain (ATCC 10876) was obtained from the American Type Culture Collection (Manassas, VA). *ΔGerI B. cereus 569* (AM1314, Tn*917*-LTV1::*gerIA5 (ino-5)* Ery^r^ *trp-1* Str^r^) and *ΔGerQ B. cereus 569* (AM1311, Tn*917*-LTV1::*gerQA2 (ino-2)* Ery^r^ *trp-1* Str^r^) strains were a generous gift from A. Moir (University of Sheffield, UK). The *B. anthracis* Sterne 34F2 strain was a generous gift from A. Casadevall (Albert Einstein College of Medicine, NY).

Spore germination was monitored on a Biomate 5 spectrophotometer at 580 nm (ThermoElectron Corporation, Waltham, MA). DPA release was monitored using published procedures [41]. Fluorescence spectroscopy was performed on an LS-50B fluorescence spectrophotometer (Perkin Elmer Life, Boston, MA,). Supernatant fractionation was performed on an Agilent 1200 HPLC system fitted with a UV-visible detector set at 340 nm (Agilent Technologies, Santa Clara, CA). Molecular weights were determined on a Thermo Finnigan LCQ ion trap mass spectrometer (ThermoFisher Scientific, Waltham, MA).

### Spore preparation

*B. cereus and B. anthracis* cells were plated in DIFCO sporulating media (DSM) (Difco Laboratories, Detroit, MI) agar at high dilutions to yield single cell clones [42]. Single *B. cereus* and *B. anthracis* colonies were replated and incubated for 72 h at 37°C. The resulting bacterial lawns were scraped from the plates and resuspended in deionized water. Spores were purified by centrifugation through a 20%–50% HistoDenz gradient. Purified spores were washed 5 times with deionized water and stored at 4°C. Spores were more than 95% pure as determined by phase-contrast microscopy.

### Analysis of inosine-mediated germination

Changes in light diffraction during spore germination were monitored at 580 nm. Spores were heat-activated at 70°C for 30 min, and resuspended in germination buffer (50 mM Tris-HCl pH 7.5, 10 mM NaCl) to an OD_580_ of 1. The spore suspension was monitored for auto-germination at OD_580_ for 1 h. Germination experiments were carried out with spores that did not autogerminate in a Biomate 5 spectrophotometer in a total volume of 1 ml. Experiments were performed in triplicate with at least two different spores preparations. Spore germination was evaluated based on the decrease in OD_580_ at room temperature. Relative OD_580_ values were expressed as a fraction of the actual OD_580_ divided by the OD_580_ obtained at the beginning of germination, and were plotted against time. All measurements showed standard deviations of less than 10%.

### Germination with conditioned supernatant

Purified spores were resuspended in 2 ml germination buffer to an OD_580_ of 1, and germination was initiated by addition of 0.2 mM inosine. Conditioned supernatants were collected following centrifugation (5,000 RPM) of germinated spores 30 min after addition of inosine. To determine heat lability and particle size of released factors, aliquots of the resulting conditioned supernatant were boiled for 30 min or filtered through a 5 kDa MWCO filter. Conditioned supernatant was then used to resuspend fresh spore aliquots. As controls, fresh spore aliquots were resuspended in 0.2 mM inosine with or without 20 µM L-alanine, and germination was monitored as described above.

To determine the effect of the inosine concentration on germination kinetics of *B. cereus* spores, supernatants were collected from spores treated with increasing inosine concentrations (0.1, 0.2, 0.4, 0.6, 0.8, and 1.0 mM: final concentration). Fresh spores (OD_580_ = 1) were germinated in the resulting conditioned supernatants. As controls, fresh spores were also germinated in 0.1, 0.2, 0.4, 0.6, 0.8, and 1.0 mM inosine in the absence of conditioned media. Germination curves were fitted using the four-parameter logistic function of SigmaPlot v.9 software to calculate the mid-time point of the germination curve (T_1/2_).

The effect of spore concentration on supernatants was tested by resuspending spores to a final OD_580_ of 0.01, 0.05, 0.1, 0.15, 0.2, 0.5, and 1. Spores were treated with 0.2 mM inosine, allowed to germinate for 30 min, and supernatants were collected from each sample and tested for their effect on germination kinetics of *B. cereus* spores, as described above.

To determine the effect of D-alanine racemase on the kinetics of inosine-mediated germination of *B. cereus*, spores were resuspended in 2 ml germination buffer to an OD_580_ of 1, and supplemented with the racemase inhibitor *D*-cycloserine (0 or 1 mM). D-cycloserine inhibits the alanine racemase, which catalyzes the conversion of active L-alanine into inhibitory D-alanine. *D*-cycloserine has been shown to potentiate L-alanine mediated germination, presumably by increasing the concentration of L-alanine available for germination. Germination was started by addition of 0.2 mM inosine and monitored 1, 2, 5, 10, 15, and 30 min post-inosine addition.

### Supernatant washout experiment

To dilute out any released germinants in the supernatant of germinated spores, 10 ml of the spore suspension (OD_580_ of 1) was added to increasing volumes of germination buffer (up to 4 l) prewarmed to 37°C containing 0.2 mM inosine and 0 or 0.04 mM alanine. As a positive control, spore suspension aliquots (200 µl) were treated with 0.2 mM inosine in the presence and absence of 0.04 mM L-alanine. Spore suspensions were incubated on a shaker at 37°C for 1 h, and then rapidly cooled to 4°C on ice. Spores and bacteria were collected from the small volumes via centrifugation at 10,000 x g. Spores and bacteria were collected from volumes above 10 ml by filtering the suspension at 4°C through a 0.2 µm PES membrane. The residue was collected from the membrane by resuspending in 2 ml germination buffer and pelleting by centrifugation at 10,000 x g. *B. cereus* pellets were smeared across a glass slide, air dried, and heat-fixed over a flame. Cells were stained using the Wirtz-Conklin staining technique, as described previously [21]. Briefly, heat-fixed spore/bacterial smears were immersed in boiling malachite green stain (5 g/100 ml water) for 1 min. Following destaining in distilled water, smears were counterstained with safranin-O (0.5 g/100 ml water) for 1 min. Smears were subsequently destained in distilled water, and mounted. *B. cereus* pellets were visualized using a Zeiss Axiophot microscope.

### Germination with dipicolinic acid (DPA)

Purified *B. cereus 569* spores were resuspended to an OD_580_ of 1 in germination buffer and germinated with 0.2 mM inosine. After 30 min, cells were centrifuged, and the concentration of released DPA in the supernatants was determined using standard protocols [43]. A solution of Ca-DPA was prepared at the same concentration (0.18 mM) present in the conditioned supernatants. Resulting solutions were supplemented with 0.2 mM inosine, and germination was monitored as above.

### Labeling of compounds released by germinating spores

Wild-type *B. cereus 569*, *ΔgerI B. cereus 569*, *ΔgerQ B. cereus 569*, and *B. anthracis* Sterne strain spores were resuspended (OD_580_ = 1) in 200 µl trimethylammonium bicarbonate buffer (TMB, pH 8.5). Wild-type *B. cereus* spores were treated with 0.2 mM inosine (in TMB) alone or 0.2 mM inosine supplemented with 0.04 mM alanine. Germination was determined as described above. After 30 min, germinated spores were pelleted by centrifugation and cell-free supernatants were collected. As a negative control, conditioned supernatant aliquots were treated with water. As positive controls, conditioned supernatants were spiked with an amino acid standard solution containing 25 µM each of L-alanine, L-arginine, L-aspartic acid, L-cysteine, L-glutamic acid, glycine, L-histidine, L-isoleucine, L-leucine, L-lysine, L-methionine, L-phenylalanine, L-proline, L-serine, L-threonine, L-tyrosine, and L-valine. A 500 µl sample of each supernatant was treated with 500 µl DMSO supplemented with 1 mM of -(3-dimethylaminopropyl)-N′-ethylcarbodiimide hydrochloride (EDAC), N-hydroxysulfosuccinimide (NHSS), and 7-amino-4-methylcoumarin (7-AMC). All reactions were incubated overnight at room temperature. After incubation, excess reagents were quenched with 1 µl glacial acetic acid for 2 h. All samples were dried under reduced pressure, re-dissolved in 100 µl of water, heated at 90°C for 30 min, and filtered through a 0.2 µm filter. Adduct fluorescence was determined on an LS-50B fluorescence spectrophotometer with excitation at 351 nm and emission at 430 nm.

### Identification of released compounds

To label amino acids in the supernatant of germinated spores we used 7-amino-4-methylcoumarin (7-AMC). Released 7-AMC adducts were separated by HPLC over a C18 reverse phase column. The mobile phase consisted of a gradient from 5% to 100% acetonitrile (MeCN in water) in 30 min. Released 7-AMC adducts were detected with a UV-visible detector set with a 340 nm cut-off filter. The identities of the amino acids present in the 7-AMC treated samples were assigned by co-elution with the similarly treated amino acid standard solution. 7-AMC adduct concentrations were determined by fluorescence spectroscopy. The identity of each 7-AMC adduct was confirmed by LCQ ion trap mass spectrometry.

### Kinetics of amino acid release

*B. cereus* spores were resuspended to an OD_580_ of 1 in 2 ml germination buffer supplemented with 0 or 1 mM *D*-cycloserine. Germination was started by addition of 0.2 mM inosine, and aliquots were collected at 0, 5, 10, 15, 20 and 30 min post-inosine addition. Aliquots were filtered sterilized and analyzed as described below.

Deuterium labeled amino acid standards (^2^H_4_-Ala) was purchased from Cambridge Isotope Laboratories (Andover, MA). Molecular biology grade isobutanol and acetyl chloride were purchased from Acros (Geel, Belgium). HPLC grade Omnisolv water and acetonitrile were purchased from EMD Chemicals Inc. (Gibbstown, NJ). An ACQUITY ultra performance liquid chromatography (UPLC) with a BEH C18 column (1.7 µm particle diameter, 2.1×50 mm) and sample organizer was used for analyte introduction. A Quattro Premier XE tandem mass spectrometer from Waters-Micromass was utilized for analyte detection.

Samples and calibration solutions were prepared for multiple reaction monitoring (MRM) quantitation of alanine following a procedure similar to that reported by Zhang *et al* [44]. Briefly, an aliquot of sample was mixed with deuterium labeled alanine and dried by vacuum centrifugation. Anhydrous isobutanolic-3 M HCl (200 µl) was added to the sample and allowed to react at room temperature for 50 min to form the isobutyl ester derivative. The reaction mixture was removed by vacuum centrifugation, and the sample was reconstituted in 200 µl of EMD water to give a final internal standard concentration of 500 nM immediately before analysis.

The sample was injected into the UPLC and run with initial solvent conditions of 20% acetonitrile and 80% water. The initial solvent mixture was maintained for 0.5 min. The solvent mixture was changed to 60% acetonitrile and 40% water in 3 min. The solvent mixture was then changed to 90% acetonitrile and 10% water in 1 min. After a 1 min hold, the solvent conditions were brought back to the original settings in 0.5 min and held for 1 min to equilibrate the column. The analyte and internal standard MRM transitions of 146>90, 146>44 and 150>94, 150>48 were monitored to calculate response factors based on peak area for quantification and confirmation. The data were processed by employing TargetLynx and MassLynx NT Software (Version 4.1, Micromass, Manchester, UK). Concentration was determined by using a calibration curve and back-calculating to reflect the original solution concentration in germination buffer.

### Determination of alanine concentration in the spore core

*B. cereus* spores were decoated following established procedures [45]. The decoated spores were lyzed by sonication in 70% acetonitrile/water. The resulting suspension was filtered-sterilized and submitted for mass spectrometry analysis as described above.
